# FALLS IN MID-LIFE: A SCOPING LITERATURE REVIEW

**DOI:** 10.1093/geroni/igz038.3156

**Published:** 2019-11-08

**Authors:** Aleksandra Zecevic, Daniella Bozzo, Alison Stirling, Helene Gagne

**Affiliations:** 1 Western University, London, Ontario, Canada; 2 Ontario Neurotrauma Foundation, Fall Prevention Community of Practice, Toronto, Ontario, Canada; 3 Ontario Neurotrauma Foundation, Toronto, Ontario, Canada

## Abstract

The implications of falls of middle-aged adults (40-64 years) on falling in late life (65+) have not received much attention. The patterns of falling over the lifespan require more research. The purpose of this scoping literature review was to answer: What is known about falls occurring in mid-life? and How falls in mid-life relate to falling over the adult lifespan? A six-stage scoping literature review framework by Levac et al. (2010) was followed. A total of 5,136 titles were identified in CINAHL, EMBASE and MEDLINE using MeSH terms for accidental fall, middle-aged and longitudinal studies. Inclusion/exclusion criteria narrowed the search to 30 full-text research articles and nine gray literature sources that were charted. Literature on falls in mid-life produced five discrete themes: two distinct populations of fallers, prevalence rates, fall-related injuries, causes of falls, and risk factors for falling. The two groups of fallers were the general population (falls prevalence 8.7-35.8%) and the special population of mid-life adults living with chronic health conditions (falls prevalence 26% for diabetes and 32.3% intellectual disabilities). Middle-aged adults had a higher proportion of injurious falls (11.5-30%) compared to older adults and extrinsic risk factors were the most frequent causes of falling (83.3%). For special populations, the risk of falls was frequently attributed to intrinsic factors. In conclusion, falls in mid-life require further exploration to establish patterns over the adult lifespan, determine influence of chronic diseases, establish clear fall incidences and risk factors, and determine if current falls prevention interventions are appropriate for mid-life adults.

